# Investigation on the Impurity Removal Behavior During the Electron Beam Melting of V-Al Alloy

**DOI:** 10.3390/ma18081710

**Published:** 2025-04-09

**Authors:** Zixin Yang, Shuaishuai Wu, Shengli Guo, Baohong Zhu, Haochen Qiu, Wei Jiang, Xuehui Yan

**Affiliations:** 1 State Key Laboratory of Nonferrous Structural Materials, China GRINM Group Co., Ltd., Beijing 100088, China; zinc0915@163.com (Z.Y.); wushuaishuai@grinm.com (S.W.); zhubh@grinm.com (B.Z.); qiuhaochen@grinm.com (H.Q.); jiangwei@grinm.com (W.J.); yanxuehui@grinm.com (X.Y.); 2 GRIMAT Engineering Institute Co., Ltd., Beijing 101407, China; 3 General Research Institute for Nonferrous Metals, Beijing 100088, China

**Keywords:** high-purity vanadium, electron beam melting, thermodynamic calculation, impurity migration

## Abstract

This study systematically investigated the behavior of impurity removal during the electron beam melting (EBM) process of V-Al alloy. Characterization techniques such as ICP, GDMS, SEM, EPMA, and TEM were used to analyze the composition content and microscopic element distribution of V-Al alloy and purified metal samples. Additionally, based on thermodynamic principles, the saturation vapor pressure and evaporation coefficients of impurity elements were calculated. The results indicate that the evaporation coefficients of Al, Fe, Co, Ni, Cr, and Ti exceed 1, enabling their effective removal during the melting process, thereby reducing their concentrations. In contrast, Si, Mo, Nb, and W exhibit evaporation coefficients significantly lower than 1, making their removal difficult. Instead, their concentrations increase due to the enrichment effect. Microstructural analysis reveals that Al migrates toward high-temperature regions, forming enrichment zones at the surface layer in contact with the electron beam. In contrast, Si, C, and O exhibit bidirectional migration characteristics, accumulating at both the upper and lower surfaces of the plate-shaped ingot. TEM observations indicate that some C reacts with V to form V_2_C, which has a higher melting point than vanadium, making further removal difficult.

## 1. Introduction

As a strategic metal, vanadium (V) is widely distributed in the Earth’s crust [1,2]. Due to its high value, vanadium and its compounds have attracted significant attention for their large-scale applications in industries such as steel production, aerospace, energy storage batteries, catalysts, chemical engineering, nuclear power, and optoelectronic materials [3,4,5,6,7]. High-purity vanadium wires can be forged into emitters for in-core self-powered neutron detectors [8,9]. In some countries, high-purity vanadium is essential for small-scale yet strategically significant applications, such as manufacturing infrared cameras and coating vanadium onto steel fuselage joints [10]. Consequently, increasing demands have been placed on the production of high-purity vanadium. The presence of various impurities in vanadium and its alloys makes achieving a high purity challenging. While high-purity vanadium can be readily produced in laboratory settings, maintaining purity and performance stability in industrial-scale production remains difficult. Therefore, developing an efficient and cost-effective method for high-purity vanadium production has become a key research focus. Among the available refining techniques, the electron beam melting (EBM) method demonstrates notable adaptability.

EBM has been demonstrated as an effective method for producing high-purity metals [11,12,13]. This technology offers several advantages, including high energy efficiency, deep penetration, rapid scanning speeds, and moderate production costs [14]. The electron beam generates an extremely high energy density (10^3^–10⁶ W/cm^2^), enabling the molten metal to reach exceptionally high temperatures, which significantly enhances purification efficiency. Compared with conventional purification methods, EBM reduces energy consumption while maintaining effectiveness. Additionally, the melting process occurs in a vacuum environment, facilitating the efficient removal of impurities and gases [15]. In 1966, Carlson [16] successfully obtained vanadium with 99.9% purity through a multistep process involving the aluminothermic reduction of V_2_O_5_, vacuum dealumination at 1973–2073 K, and subsequent EBM purification. In this study, Al and O are impurities that are difficult to remove. Although Al can be volatilized in a vacuum at 1973 K, it is difficult to reduce its content to an extremely low level. O is tightly bound to vanadium. During the de-aluminization process, some O can be removed along with the volatilization of Al. However, to reduce the content of O to an extremely low level, parameters such as the temperature, time, and vacuum degree of melting need to be precisely controlled. Schmidt et al. [17] developed a method for producing high-purity vanadium with a low oxygen and silicon content by integrating aluminothermic reduction, EBM purification, and calcium deoxidation. During the reaction process, Fe forms a relatively stable alloy structure with vanadium. During the electron beam melting process, although a certain effect has been achieved in removing a high content of Fe, it is still difficult to completely remove it. This makes it necessary to use higher energy and more complex process conditions to remove Fe. Peng [18] achieved 99.7% vanadium purity by employing a two-stage EBM process using V-Al alloys in an electron beam cold-bed melting furnace. It was found in this study that VN is difficult to decompose and melt, and the content of N gradually increases as the EBM progress. Due to the relatively stable chemical properties of Si in vanadium, it is difficult to completely remove Si with existing processes. In the final product, the content of Si is relatively high. Despite these advancements, the impurity removal mechanisms and migration behaviors during the EBM process of vanadium remain poorly understood. In particular, the effect of EBM power on the migration of impurity elements in metallic vanadium has not been thoroughly investigated.

In this study, the migration behavior of impurity elements during the EBM process of V-Al alloy was investigated. The microscopic morphologies and composition of slab casting after melting were analyzed to elucidate the migration patterns of impurity elements and the effect of melting power on their removal behavior. Additionally, a theoretical model of impurity removal was established to predict impurity removal trends, providing a theoretical foundation for the extraction of high-purity vanadium through EBM.

## 2. Materials and Methods

**Figure 1:**
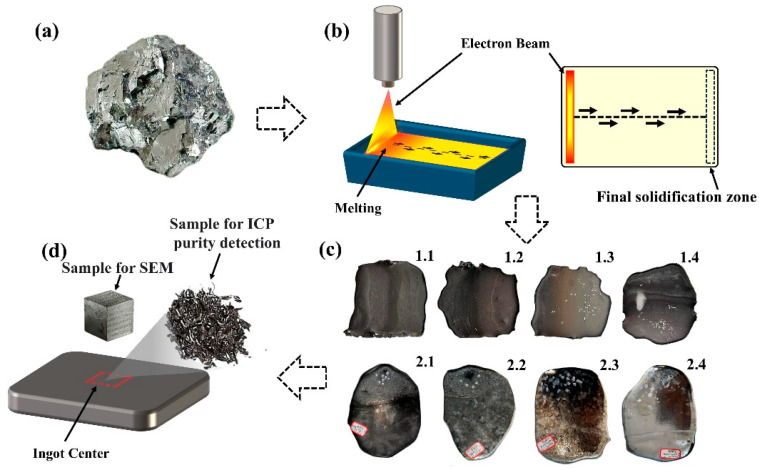
Raw material and slab casting after EBM: (**a**) V-Al alloy raw material used for the experiment; (**b**) EBM process and the straight scanning path employed during the process; (**c**) slab casting obtained after two melting sessions; (**d**) sampling locations of chips and samples for compositional analysis.

The V-Al alloy was synthesized via the aluminothermic reduction method using aluminum powder and V_2_O_5_, as shown in Figure 1a. The actual composition of the alloy is presented in Table 1. Prior to melting, the raw materials were ultrasonically cleaned in industrial ethanol, thoroughly dried, and accurately weighed using electronic scales to ensure precise measurements.

The EBM experiment (EB-300) was conducted using a 300 kW EBM furnace (Shenyang Science Instrument Co., Ltd. of the Chinese Academy of Sciences, Shenyang, China). The electron beam gun operated at an accelerating voltage of 30 kV, and the melting process was performed in a water-cooled copper crucible. During the experiment, the vacuum levels in the smelting chamber and electron gun chamber were maintained at 5 × 10^−2^ Pa and 1 × 10^−3^ Pa, respectively. The electron beam irradiated the material’s surface along a straight scanning path, as illustrated in Figure 1b. The parameters for the two melting processes are detailed in Table 2 and Table 3, while the slab castings obtained after melting are shown in Figure 1c.

Samples with a length and width of 10 mm and a thickness equal to that of the slab casting were cut from the casting billet, whose surface was not polished; then, the samples were ground successively with #240, #400, #600, #800, #1200, #1600, and #2000 sandpapers and polished with diamond polishing paste for the analysis of the microstructure. After polishing the surface of each slab casting billet, 30 g of filings was collected for inductively coupled plasma spectrometry. Microstructural observations were conducted using a JXA-8530F Electron Probe Microanalysis (EPMA, for JEOL Ltd., Tokyo, Japan) and a Zeiss Sigma-300 scanning electron microscope (SEM, for Carl Zeiss AG, Oberkochen, Germany) equipped with an energy-dispersive spectrometer (EDS). An ICAP–700 inductively coupled plasma spectrometer (ICP, for Thermo Fisher Scientific, Waltham, MA, USA) was used to test the elemental content in the raw materials, and the measurement accuracy was more than 0.0001%. The composition of the eight melted samples was analyzed using a GDMS-2000 glow discharge mass spectrometer (GDMS, for Ametek, Inc., Berwyn, MA, USA); the measurement range of the element content can be accurate to less than 0.01%. An HCS-500 Carbon and Sulfur Analyzer (for Wuxi Yingmaiji Dongda Machinery Technology Co., Ltd., Wuxi, China) was used to measure the carbon content in the samples after smelting; the lower limit of measurement can reach 0.0001%. Vanadium chips were extracted from the center of the slab castings after the surface oxide layer was removed by polishing, as shown in Figure 1d. Each sample was tested three times to obtain an average value.

## 3. Results and Discussion

### 3.1. Raw Material Analysis

**Figure 2:**
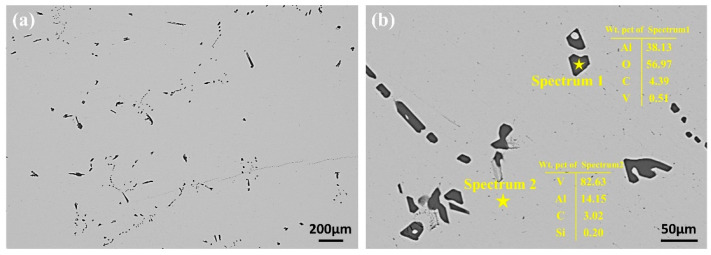
SEM results for the V-Al alloy alloy: (**a**) low-magnification SEM image of the V-Al alloy alloy, showing the overall structure; (**b**) EDS point scan results of the impurity phase and matrix, highlighting the elemental composition.

To determine the forms of impurities present in the raw materials before EBM, an analysis of the V-Al alloy was conducted. Figure 2 presents the SEM micrographs of the V-Al alloy along with the energy spectrum analysis of the matrix and impurity phases. As shown in Figure 2a, the V-Al alloy exhibited two regions with distinct contrasts under the scanning electron microscope. To investigate the composition of these regions, a local area was magnified, and the elemental compositions of different phases were analyzed. The EDS energy spectrum scanning results at points 1 and 2 are displayed in Figure 2b. The analysis revealed that the matrix primarily consisted of V, with small amounts of Al, Si, and C. The rod-like phase was predominantly composed of Al and O, with an atomic ratio of approximately 2:3 for Al and O. Based on this ratio, the phase was preliminarily identified as Al_2_O_3_.

**Figure 3:**
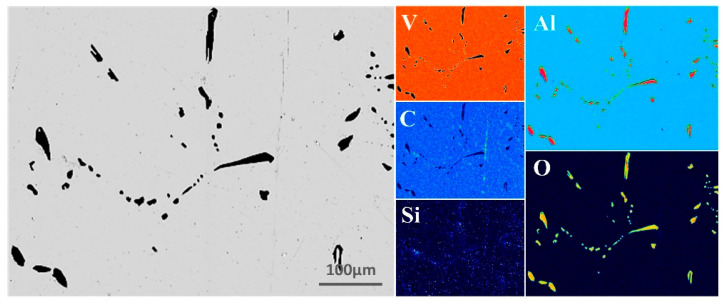
EPMA micrographs of V-Al alloy.

During the EBM of the V-Al alloy into high-purity vanadium, particular attention was given to elements such as Al, Si, C, and O. To intuitively and clearly visualize the elemental distribution in the V-Al alloy, EPMA surface scanning was performed, and the results are presented in Figure 3. The analysis indicates that V constitutes the matrix of the material, while Al and O are primarily concentrated in the impurity phase and are nearly absent in the matrix, suggesting that Al predominantly exists in the form of oxides. Additionally, there is a small amount of C element distributed in the vanadium matrix. Based on the current analysis results and relevant theoretical speculations, C may be uniformly dispersed in the lattice interstices of the vanadium matrix in the form of trace solid solutions, or it may combine with vanadium atoms to form carbides, thus existing in the structure of the vanadium matrix. The Si content in the vanadium matrix is minimal.

**Figure 4:**
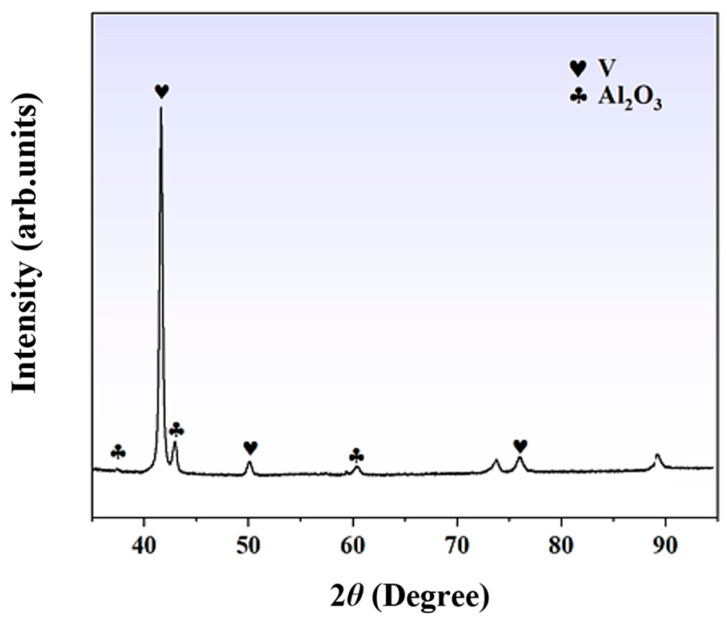
X-ray diffraction pattern of the V-Al alloy.

X-ray diffraction (XRD) analysis was performed to examine the physical phases of the V-Al alloy, and the corresponding diffraction pattern is presented in Figure 4. The diffraction results reveal distinct BCC-V structural characteristic peaks in the raw material, which are consistent with the aforementioned compositional analysis. Additionally, relatively weak but identifiable Al_2_O_3_ diffraction peaks can be observed, indicating the presence of alumina in the raw material. The XRD analysis confirms that the primary phases of the V-Al alloy are BCC-V and Al_2_O_3_. To achieve high-purity vanadium from the V-Al alloy, a significant amount of Al must first be removed to obtain vanadium of primary purity. Subsequently, a more refined secondary smelting process is required to further purify the material.

### 3.2. Changes in Impurity Content

GDMS was employed to analyze the samples after melting. Table 4 and Table 5 detail the concentrations of various elements in the different melting conditions.

During primary melting, vanadium purity increased from 99.67% to 99.79% as the melting power rose from 40 kW to 55 kW, demonstrating that higher power enhances volatile element separation. In secondary smelting, the highest purity (99.95%) was achieved at 50 kW, where most impurities were removed without excessive concentration of Si, W, and Mo. Therefore, 50 kW is the optimal melting power for obtaining high-purity vanadium with stable elemental concentrations.

Based on the tabulated data, the concentrations of Al and Fe decrease significantly during the EBM process. While the concentrations of Ni, Co, Nb, and Ti also decrease, their reduction is notably less pronounced compared to that of Al and Fe. In contrast, the concentrations of Si, Mo, W, and Nb exhibit an increasing trend rather than a reduction as the EBM power increases.

### 3.3. Thermodynamic Analysis of Inclusion Removal

#### 3.3.1. Thermodynamic Theoretical Analysis of First Melting

Electron beam melting typically occurs at extremely high temperatures, making it challenging to directly monitor the temperature of the molten pool. Due to the high-vacuum environment during EBM, even when an infrared thermometer is employed, the transmission characteristics of infrared radiation will be altered, leading to deviations in the temperature measurement results. Moreover, clarifying the relationship between the EBM power and the actual melting temperature serves as the foundation for an in-depth exploration of the EBM mechanism and the physical and chemical processes of materials. Therefore, the relationship between the melting power and the actual melting temperature is established through theoretical calculations.

Since the purity of metallic vanadium exceeds 99% during the second EBM process and the impurity content is relatively low, the mass loss during the EBM process can be approximately regarded as the loss of the matrix vanadium. The theoretical maximum evaporation rate of vanadium during EBM can be expressed as follows:

(1) $$V_{V}={\left(\frac{M_{V}}{2\pi \mathrm{RT}}\right)}^{\frac{1}{2}}\cdot P_{V}^{0}$$

where V_V_ represents the theoretical maximum volatilization rate of vanadium (g/m^2^s), M_V_ represents the atomic mass of vanadium, T represents the temperature of the melt surface (K), P_V_^0^ represents the saturated vapor pressure of pure vanadium (Pa), and R is the gas constant with a value of 8.314 J/(K·mol). The relationship between the saturated vapor pressure of vanadium and the temperature, T, is as follows [19]:

(2) $${\mathrm{lgP}}_{V}=\frac{26900}{T}+10.12+0.33\mathrm{lgT}-2.65\times 10^{-4}T$$

Assuming that the volatilization rate of vanadium during the EBM process is constant, then:

(3) $$V_{V}=\Delta m_{V}/\mathrm{At}$$

where A represents the surface area of the melt (m^2^). According to the length and width of the plate-shaped casting blank after EBM, the maximum surface area of the melt during the evaporation of the plate-shaped casting blank can be calculated as 0.0285 m^2^. t represents the EBM time (s). By combining Equations (1)–(3), an equation containing only the unknown T is obtained, and then the temperature of the surface of the molten pool during EBM can be derived. Combined with the mass losses after actual smelting in Table 2 and Table 3, the melting temperatures were calculated as follows: 3202 K at 40 kW, 3267 K at 45 kW, 3326 K at 50 kW, and 3344 K at 55 kW.

#### 3.3.2. Thermodynamic Theoretical Analysis of First Melting

**Figure 5:**
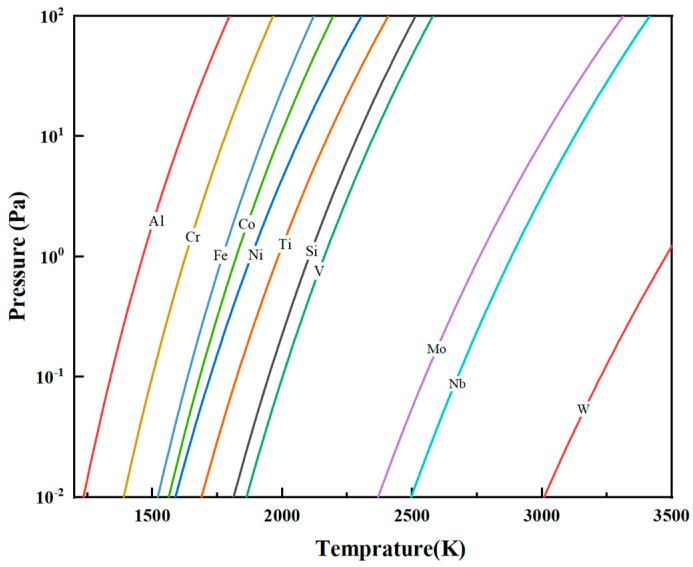
Saturation vapor pressure versus temperature for vanadium and its common impurity elements.

The primary mechanism for removing impurities during the EBM process is based on the differences in the vapor pressures of various impurity elements and vanadium at high temperatures. The saturation vapor pressure serves as the fundamental criterion for determining whether impurities can be effectively removed. The vapor pressure of each element at a given temperature can be described using the Clausius–Clapeyron Equation [19]:

(4) $$\log p=-\frac{A}{T}+B+C\log T+10^{-3}DT$$

where *A*, *B*, *C*, and *D* are constants specific to each element and *T* is the thermodynamic temperature (K). By substituting the common impurities in vanadium and the *A*, *B*, *C*, and *D* values of vanadium [20] into Equation (4), the saturation vapor pressure–temperature curves for V and its impurities were determined (Figure 5). All elements of saturation vapor pressures increase with temperature, but their rates of increase vary. Mo, Nb, and W, with lower pressures than V, are refractory and hard to volatilize. The saturation vapor pressure of Si is close to that of V, and it is not easily removed. Fe, Al, Cr, Ni, Co, and Ti, with higher pressures, are more volatile.

As a function of temperature, saturation vapor pressure increases with rising temperature. With the progressive increase in EBM power, the melt temperature correspondingly rises, leading to an increase in the saturation vapor pressures of various elements and enhancing their tendency to volatilize. During the primary melting stage of the V-Al alloy, elements such as Fe, Al, Cr, Ni, Co, and Ti exhibit saturation vapor pressures higher than that of vanadium. At elevated temperatures, these elements show greater volatilization tendencies, continuously evaporating from the alloy phase into the gaseous phase, thereby reducing their concentrations in the final material. In contrast, Mo, Nb, and W, which have lower saturation vapor pressures than vanadium, remain non-volatile, resulting in no significant reduction in their contents after melting.

#### 3.3.3. Thermodynamic Theoretical Analysis of Second Melting

The volatilization process of an element generally consists of the following three steps: the element migrates from within the metal or alloy melt through the liquid-phase boundary layer to the melt surface; the element volatilizes at the melt surface; and the volatilized species are transported through the gas-phase boundary layer into the gas phase.

Comparing saturation vapor pressures alone only provides a theoretical indication of whether EBM can remove particular impurities. To further clarify the actual removal trends and the effect of the electron beam on these impurities in high-purity metals, the evaporation behavior of impurities in the vanadium melt can be characterized by the evaporation coefficient, *β*. *β* < 1 indicates that the impurity cannot be effectively removed by EBM, *β* > 1 suggests that it can be removed, and *β* > 10 signifies significant removal of the impurity.

The total amount of common impurities in pure vanadium is less than 1000 µg/g, allowing the vanadium melt to be treated as a dilute solution. In this context, vanadium (the solvent) is assumed to follow Raoult’s Law, with its activity coefficient taken as 1, while each impurity (the solute) follows Henry’s Law, with a constant Henry’s activity coefficient. Accordingly, the evaporation coefficient for removing impurities from a vanadium melt can be expressed as follows [21]:

(5) $$\beta =\sqrt{\frac{M_{j}}{M_{i}}}\cdot \frac{\gamma_{i}p_{i}^{*}}{p_{j}^{*}}$$

where *M_i_* and *M_j_* (g/mol) are the molar masses of *i* and *j*; *γ_i_* is the activity coefficient of the solute, *i*; and p_i_^*^ and *p_j_* (Pa) are the saturated vapor pressures of *i* and *j*. The infinite-dilution activity coefficient of component *i* in a binary alloy at any temperature can be expressed as shown in Equation (6) [22]:

(6) $$\ln\gamma_{i}^{\infty }=\frac{\left[1-\frac{1}{14}T\left(\frac{T_{mi}+T_{mj}}{T_{mi}\cdot T_{mj}}\right)\right]\cdot f_{ij}[1+\mu_{i}x_{j}(\varphi_{i}-\varphi_{j})]}{RTV_{j}^{2/3}}$$

where [23]

(7) $$f_{ij}=\frac{2pV_{i}^{2/3}V_{j}^{2/3}[q{\left(\Delta n_{\mathrm{ws}}^{1/3}\right)}^{2}/p-{\left(\Delta \varphi \right)}^{2}-a(r/p)]}{{\left(\Delta n_{\mathrm{ws}}^{1/3}\right)}_{i}^{-1}+{\left(\Delta n_{\mathrm{ws}}^{1/3}\right)}_{j}^{-1}}$$

where $x_{j}$ is the mole fraction of the matrix (*j*); *T_mi_* and *T_mj_* are the melting points of elements *i* and *j*;${\;V}_{i},$ and $V_{j}\;$are the molar volumes of elements i and j ;$\;{\left(n_{ws}\right)}_{i\;}$ and ${\;\left(n_{ws}\right)}_{j}\;$are their corresponding electron densities; and${\;\varphi }_{i\;}$and${\;\varphi }_{j\;}$denote the electronegativities of elements i and j, respectively. The parameters *p*, *q*, *u*, *a*, and *r/p* are fixed values.

**Figure 6:**
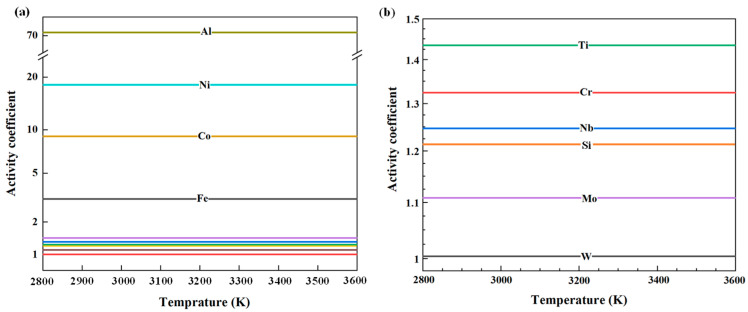
Variation in impurity activity coefficients in vanadium melts as a function of temperature: (**a**) Al, Ni, Co, and Fe; (**b**) Ti, Cr, Nb, Si, W, and Mo.

Substitute the mole fraction of the matrix after secondary smelting and the relevant parameters [23] into Equations (6) and (7).Under the condition of infinite dilution, the activity coefficients of various impurity elements in pure vanadium can be determined as functions of the melt temperature. The corresponding results are shown in Figure 6.

**Figure 7:**
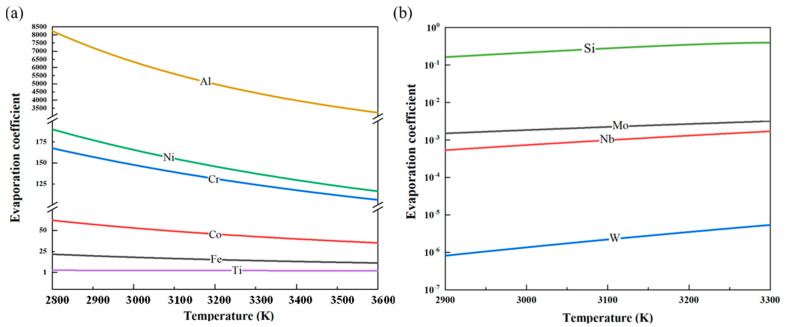
Variation in the evaporation coefficients with temperature for each impurity element in vanadium: (**a**) Al, Ni, Co, Cr, Fe, and Ti; (**b**) Si, Mo, Nb, and W.

After computing the temperature-dependent activity coefficients of each impurity element, by using Equation (2), the curves depicting the variation in the corresponding evaporation coefficients of these elements in vanadium with temperature were obtained, as shown in Figure 7.

During the electron beam melting (EBM) process, Al exhibits an evaporation coefficient greater than 10, significantly reducing its content in the vanadium matrix. Similarly, Ni, Cr, Co, and Fe also have high evaporation coefficients, enabling their effective removal through EBM. In contrast, Ti and Co have evaporation coefficients close to 1 but less than 10, resulting in suboptimal removal efficiency. Meanwhile, Si, Mo, Nb, and W possess evaporation coefficients far below 1, rendering them nearly non-volatile. As melting power increases, the vapor pressure of the vanadium matrix rises, leading to increased volatilization of vanadium. In this process, although the relative proportions of non-volatile elements (Si, W, Nb, and Mo) appear to increase, this effect primarily results from the preferential evaporation of more volatile elements, leading to a concentration effect.

### 3.4. Microstructure Evolution

#### 3.4.1. Analysis of the First Melting

**Figure 8:**
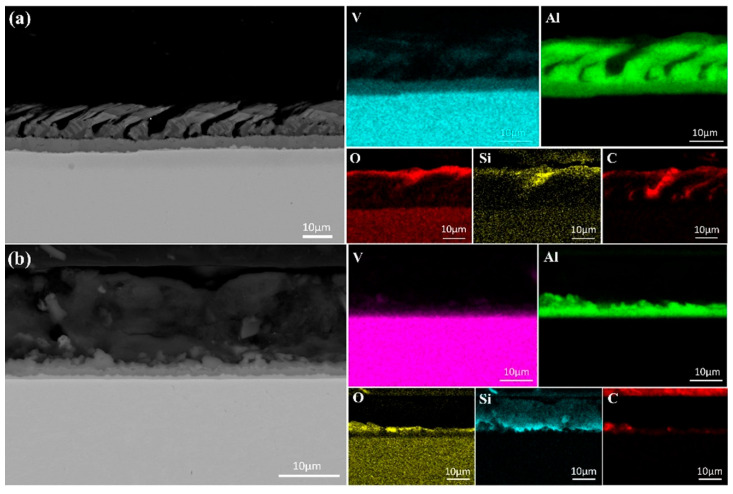
SEM micrographs and surface element distributions at the top of the specimens after first melting: (**a**) 40 kW; (**b**) 55 kW.

Figure 8 presents the SEM images and elemental face scan distributions at the top of samples melted at 40 kW and 55 kW. A pure Al layer formed on each sample’s surface, indicating that alumina in the V-Al alloy decomposed during EBM. Al_2_O_3_ decomposes into Al, Al_2_O, and AlO above 1700 K [24]. AlO, though transiently formed at high temperatures, is unstable and converts into other oxides, such as Al_2_O_3_ and Al_2_O [25]. Al_2_O is highly volatile [26], and its gas phase, produced from Al_2_O_3_ decomposition, rises and volatilizes at the melt surface. With a low melting point of 933.47 K, Al gains high kinetic energy at over 2000 K in EBM, enabling migration from the melt-pool interior to the surface, driven by high-temperature diffusion and melt-pool convection.

Further analysis revealed that the thickness of the Al-rich layer decreased with increasing melting power, measuring approximately 20 μm at 40 kW and only 5 μm at 55 kW. This indicates that higher power accelerates evaporation of Al. However, the presence of an Al-rich layer at 55 kW suggests that a single melting step is insufficient for achieving high-purity vanadium, necessitating additional melting cycles. In addition to the Al-rich layer, regions enriched in O, C, and Si were observed at the sample surface. This indicates that, along with Al migration, small amounts of C, Si, and O in the matrix also moved due to temperature and chemical potential gradients within the melt pool. The partial overlap of Si and O distributions suggests the formation of silicon oxides at the surface.

Notably, the Si-rich layer thickness increased from 5 μm at 40 kW to approximately 15 μm at 55 kW, indicating that higher melting power facilitates Si migration from the matrix to the surface. With a density of 2330 kg/m^3^, lower than that of V and Al, Si may rise due to buoyancy in the molten metal. During melting, convection and melt stirring further transport lighter Si atoms to the upper regions, leaving the vanadium-rich matrix with fewer impurities. Although some Al remains near the Al-rich layer, its concentration decreases with distance, resulting in purer vanadium.

**Figure 9:**
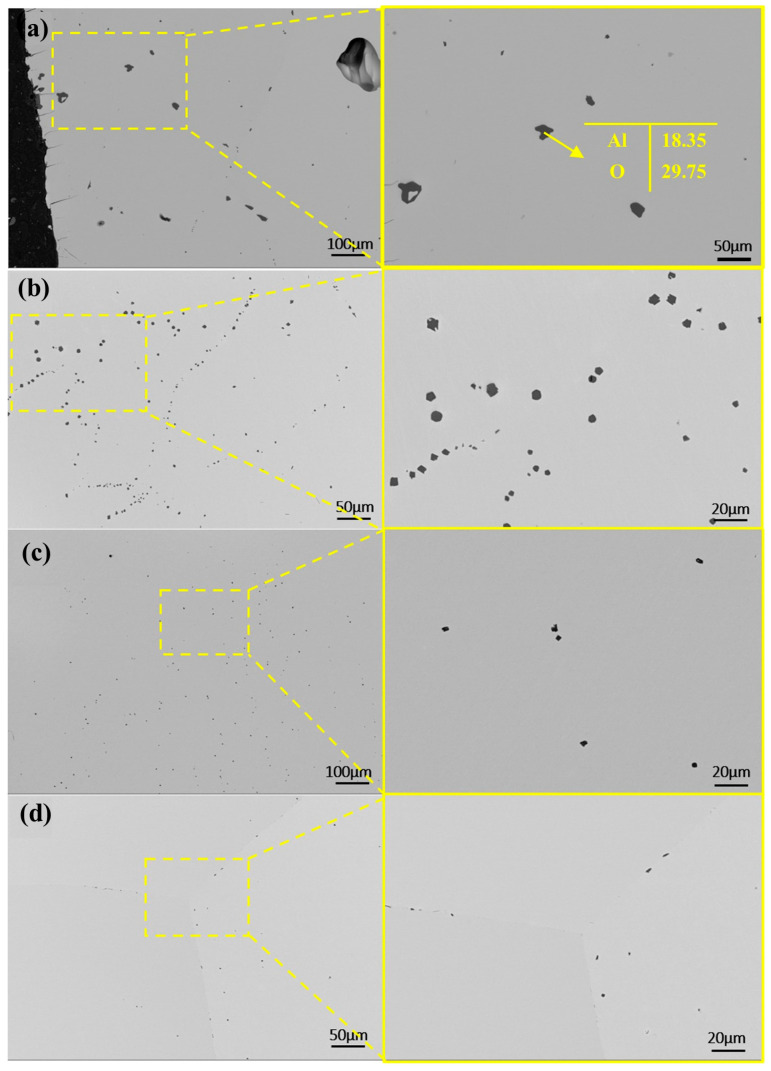
SEM micrographs and EDS in the central regions of samples after first melting at different powers: (**a**) 40 kW; (**b**) 45 kW; (**c**) 50 kW; (**d**) 55 kW.

As shown in Figure 9a, the SEM image of the sample’s mid-section after a single melting step reveals that residual impurities are primarily alumina, as confirmed by point scanning. Compared to the raw material, which exhibits widespread and uniformly distributed alumina impurities, the electron beam-melted sample contains significantly fewer internal impurities. However, additional melting cycles are required for complete removal.

With increasing melting power, impurities in the samples decreased in both number and size. In Figure 9a, at a lower power, dispersed impurities measured around 25 μm. At 45 kW, large impurities disappeared, replaced by smaller ones. Higher power promotes impurity fragmentation and dispersion. At elevated temperatures, thermal stress may break large impurities into smaller particles, while stronger melt-pool stirring enhances their uniform distribution.

**Figure 10:**
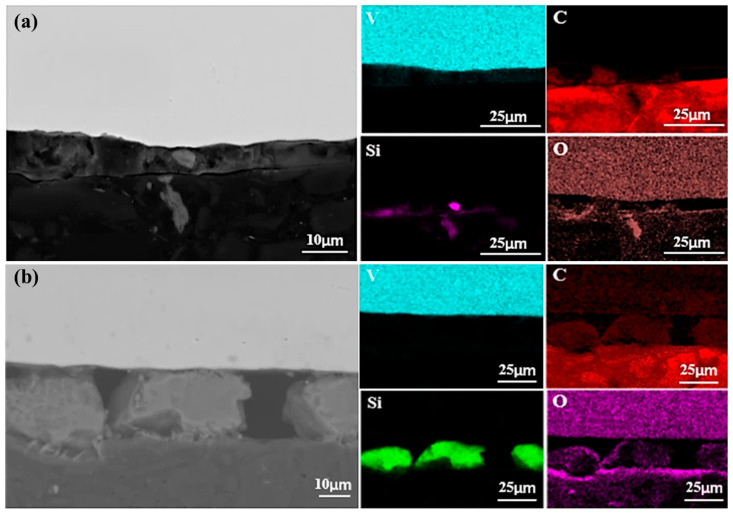
SEM micrographs of the bottom surface of samples (in contact with the cooling water) after first melting: (**a**) 40 kW; (**b**) 55 kW.

Figure 10 presents SEM images of the sample bottom after a single melting. Elemental analysis indicates that Si and C migrated downward, while Al did not. This is attributed to Al’s lower melting point (933 K) compared to Si (1687 K), causing Al to liquefy earlier and be more readily pushed upward by buoyancy in the melt. The overlap of C and O in Figure 10 suggests the formation of carbon oxides during melting. As the melting power increases, the carbon oxide content decreases, likely due to enhanced decomposition at higher power levels.

The migration and deposition of Si are particularly notable. At 40 kW, Si formed only a minor aggregation at the sample bottom, whereas at 55 kW, it developed a continuous deposition layer approximately 20 μm thick. This suggests that higher power facilitates Si accumulation at the bottom, forming a significant layer. Additionally, Si also migrates upward, as observed at the sample top. These findings indicate that migration of silicon during melting is bidirectional, occurring both toward the bottom, where the melt contacts the cooling water, and toward the top.

**Figure 11:**
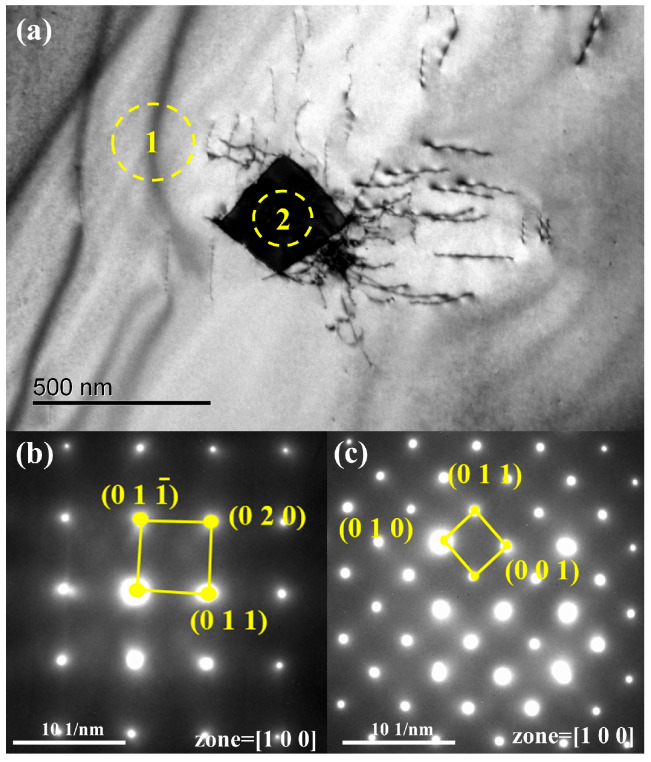
TEM micrographs of the samples after a single melting: (**a**) the area for analysis; (**b**) electron diffraction pattern of region 1 (vanadium); (**c**) electron diffraction pattern of region 2 (V_2_C).

**Figure 12:**
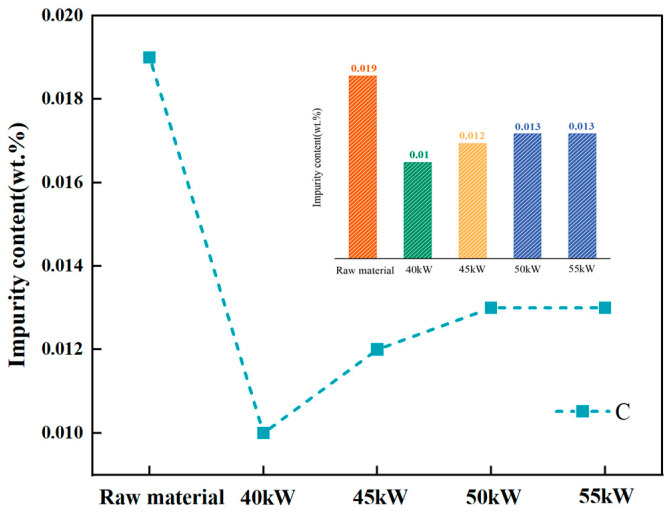
Content of C before and after first melting.

An approximately 250 nm bulk phase is visible in Figure 11. Figure 11a,b show diffraction spots for the matrix (region 1) and the bulk phase (region 2). The pattern in Figure 11a corresponds to vanadium, while Figure 11b matches V_2_C. V_2_C has a low decomposition pressure and a high melting point (2437 K) [27]; the melting point of V_2_C is more than 523 K higher than that of vanadium (2183 K), and the decomposition pressure of V_2_C is very low. It is difficult for V_2_C to dissociate in a vacuum state [18]. It is difficult to remove it through vacuum smelting. As the vanadium matrix volatilizes, carbon becomes more concentrated, increasing the overall carbon content, as shown in Figure 12.

#### 3.4.2. Analysis of the Secondary Melting

**Figure 13:**
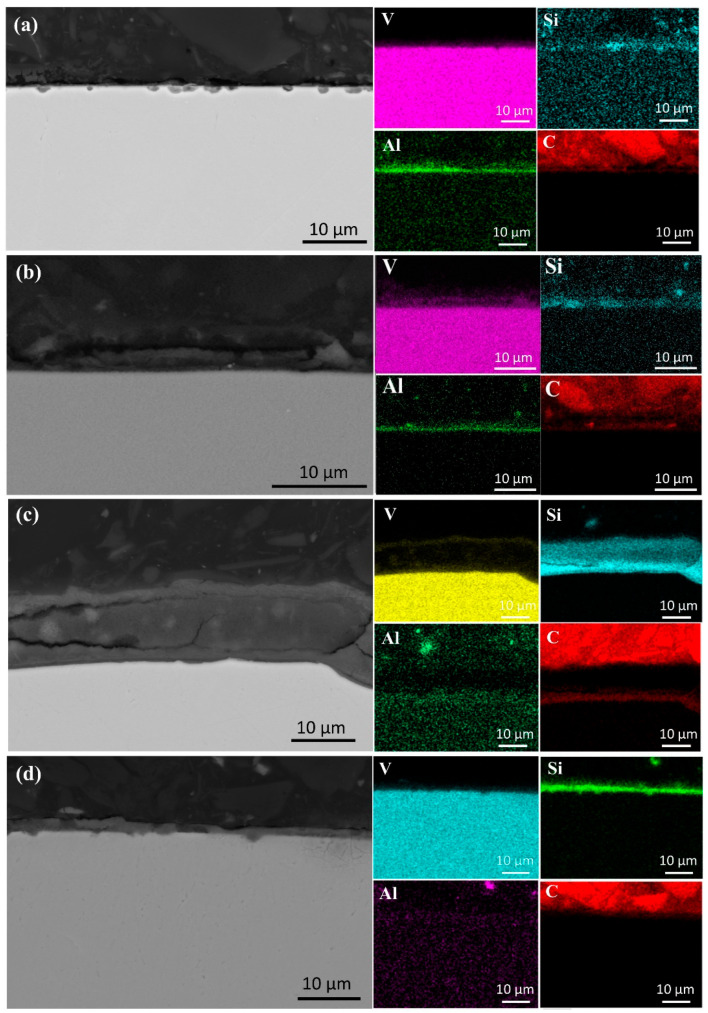
SEM micrographs and surface element distributions at the top of the specimens after first melting: (**a**) 40 kW; (**b**) 45 kW; (**c**) 50 kW; (**d**) 55 kW.

As shown in Figure 13, after secondary melting, only the samples melted at 40 kW and 45 kW exhibited a trace aluminum-enriched layer, approximately 1 μm thick, at the top. In these samples, a 1 μm thick Si-containing layer was also present, with less aggregation at a lower power. As the power increased to 50 kW, the silica-rich layer thickened to 10 μm, indicating that higher power enhanced Si migration and enrichment. However, at 55 kW, this layer thinned to 3 μm, likely due to elevated temperatures and rapid melt flow promoting Si diffusion, reducing local enrichment and potentially affecting metal purity.

Si deposition at the sample bottom follows a similar trend. At 40 kW, the Si layer measured 2 μm, increasing to 10 μm at 50 kW, then thinning to 5 μm at 55 kW. This suggests an optimal power range for Si precipitation and deposition. Beyond this range, Si redistributes within the matrix rather than aggregating. Therefore, melting power plays a critical role in elemental distribution and deposition, necessitating precise power control to optimize element migration and achieve higher-purity vanadium.

**Figure 14:**
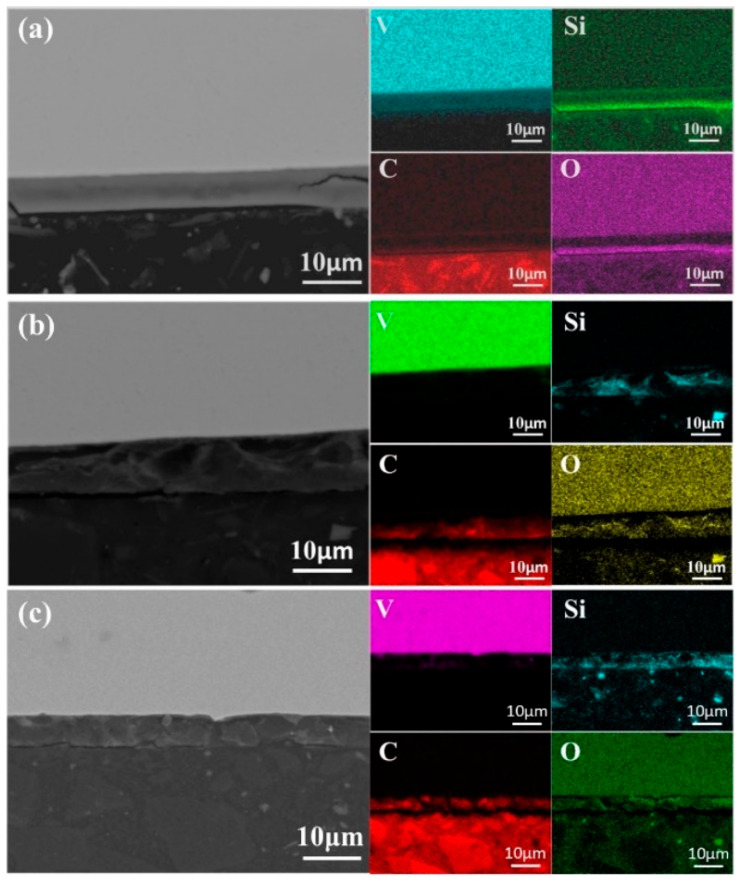
SEM micrographs of the bottom region of samples after second melting: (**a**) 45 kW; (**b**) 50 kW; (**c**) 55 kW.

**Figure 15:**
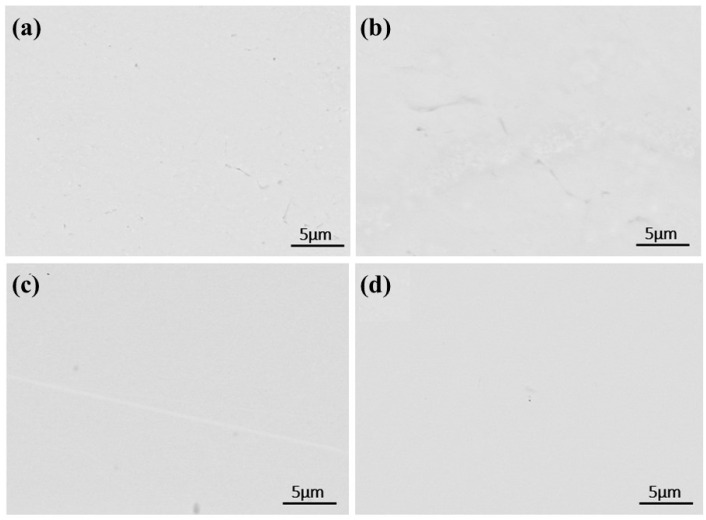
SEM micrographs of the central region of samples after second melting: (**a**) 40 kW; (**b**) 45 kW; (**c**) 50 kW; (**d**) 55 kW.

As shown in Figure 14, bottom-scanning images reveal overlapping regions of Si, O, and C. After primary melting, only C and O overlap, indicating the formation of carbon oxides. Following secondary melting, these phases likely transform into carbosilicides, silicon oxides, and carbon oxides. Figure 15 presents a backscattered image of an uncorroded sample. Unlike the once-melted sample, no dispersed aluminum oxide impurities can be observed in the matrix. Each melting step contributes to impurity removal. During the first melting, large alumina particles break down, releasing O and Al atoms, though some alumina and other impurities remain. With additional melting cycles, persistent impurities gradually diminish, facilitating improved solid-phase impurity separation, reduced microscopic segregation, enhanced metal homogeneity, and higher-purity liquid-phase metal.

## 4. Conclusions

(1) In the raw V-Al alloy, Al primarily exists in the form of Al_2_O_3_, while the main impurity elements include Al, Si, Fe, C, and O. C is distributed within the vanadium matrix. To obtain high-purity vanadium, a significant amount of Al must first be removed, followed by secondary melting.

(2) Based on the evaporation characteristics of the materials, a thermodynamic analysis incorporating the saturation vapor pressure, activity coefficient, and evaporation coefficient was conducted to evaluate impurity content variations after melting. The results indicate that Si, Mo, Nb, and W exhibit lower saturation vapor pressures than V, with evaporation coefficients significantly below 1, making their removal difficult. Therefore, the content of these elements should be strictly controlled in the raw materials to reduce it at the source. For these impurities that are difficult to volatilize, other refining methods may be required, such as chemical separation, electrolytic refining, or zone melting, in the future. In contrast, Al, Fe, Ni, Co, Cr, and Ti have higher saturation vapor pressures than V. Meanwhile their evaporation coefficients are above 1, facilitating their effective removal.

(3) During primary melting, a pure Al layer forms at the sample top, with its thickness decreasing as the power increases. Simultaneously, Si, O, and C migrate bidirectionally due to temperature and chemical potential gradients, accumulating at both the top and bottom. The carbon oxide content decreases with increasing power, while the number and size of impurities in the middle region also reduce.

(4) In secondary melting, Si enrichment at the top and bottom varies with power. At 40–45 kW, a thin Si layer and an extremely thin Al-rich layer are present at the top. At 50 kW, the Al-rich layer disappears and the Si layer thickens. At 55 kW, the Si layer becomes thinner, with a similar trend observed at the bottom. This indicates the existence of an optimal melting power range, beyond which Si remelts into the matrix.

(5) Multiple melting cycles help reduce impurities and improve metal purity. During primary melting, vanadium purity increases with power; the highest purity (99.79%) is achieved at 55 kW. In secondary melting, the highest purity (99.95%) is achieved at 50 kW.

## Figures and Tables

**Table 1:** Chemical composition of V-Al alloy.

Element	V	Al	Si	Fe	Mo	Cr
**wt. %**	84.58	15.19	0.035	0.024	0.001	0.0024
**Element**	**Nb**	**Ni**	**W**	**C**	**N**	**O**
**wt. %**	<0.0005	<0.0005	0.002	0.019	0.026	0.12

**Table 2:** Parameters related to the first melting.

Serial Number	Melting Power (kW)	Melting Time (s)	Mass Before Melting (kg)	Mass After Melting (kg)	Mass Loss Rate (%)
1.1	40	709	2.5	2.05	18.0
1.2	45	723	2.5	2.04	18.4
1.3	50	715	2.5	2.01	19.6
1.4	55	727	2.5	1.92	23.2

**Table 3:** Parameters related to the second melting.

Serial Number	Melting Power (kW)	Melting Time (s)	Mass Before Melting (kg)	Mass After Melting (kg)	Mass Loss Rate (%)
2.1	40	762	2.05	1.84	10.2
2.2	45	776	2.04	1.76	13.7
2.3	50	781	2.01	1.75	12.9
2.4	55	759	1.92	1.66	13.5

**Table 4:** Elemental content in samples at different melting powers after first melting.

		Power	40 kW	45 kW	50 kW	55 kW
	Content (ppm)					
Element						
**Al**			2800	2300	2200	1700
**Si**			260	280	290	290
**Fe**			160	120	79	56
**W**			31	31	33	33
**Mo**			12	13	13	13
**Cr**			6.1	1.7	0.39	0.044
**Nb**			2	2.3	2.3	2.4
**Ti**			2.6	2.4	2.3	2.3
**Co**			0.2	0.16	0.16	0.15
**Ni**			5.3	4.2	4.1	3.9
**Total Amount of Impurities**			3279	2755	2624	2101
**Purity (wt.%)**			99.67%	99.72%	99.74%	99.79%

**Table 5:** Elemental content in samples at different melting powers after second melting.

		Power	40 kW	45 kW	50 kW	55 kW
	Content (ppm)					
Element						
**Al**			2100	1800	110	200
**Si**			280	290	300	310
**Fe**			91	44	23	20
**W**			36	38	40	42
**Mo**			13	14	16	19
**Cr**			0.9	0.088	0.041	0.012
**Nb**			2.5	2.5	2.8	5.1
**Ti**			2.4	2.1	1.9	1.8
**Co**			0.16	0.12	0.087	0.1
**Ni**			4	2.9	2.1	2
**Total Amount of Impurities**			2530	2194	494	600
**Purity (wt.%)**			99.75%	99.78%	99.95%	99.94%

## Data Availability

The original contributions presented in this study are included in the article. Further inquiries can be directed to the corresponding author.
